# Emerging Contaminant Imidacloprid in Mediterranean Soils: The Risk of Accumulation Is Greater than the Risk of Leaching

**DOI:** 10.3390/toxics10070358

**Published:** 2022-06-30

**Authors:** Mirna Petković Didović, Tomasz Kowalkowski, Dalibor Broznić

**Affiliations:** 1 Department of Medical Chemistry, Biochemistry and Clinical Chemistry, Faculty of Medicine, University of Rijeka, 51000 Rijeka, Croatia; mirnapd@uniri.hr; 2 Environmental Chemistry and Bioanalytics, Faculty of Chemistry, Nicolaus Copernicus University, Gagarina 7, 87-100 Toruń, Poland; tomasz.kowalkowski@umk.pl

**Keywords:** emerging pollutant, neonicotinoids, soil health, sorption, leaching, hematite

## Abstract

Imidacloprid (IMI) is an extensively used neonicotinoid insecticide whose occurrence in the environment is a worldwide problem. Its sorption/transport properties are recognized as one of the key knowledge gaps hindering policymaking regarding its international routine monitoring in soils. Therefore, we studied IMI transport behaviour in Croatian Mediterranean soils using column experiments. Breakthrough curves were analysed using the two-site adsorption model and compared against dimethoate (DIM). Transport parameters were correlated to soil physicochemical properties. The results indicate that IMI shows a high degree of preference for soil organic matter over any other soil constituent. For IMI, the clay did not exhibit any sorption activity, while hematite did act as an active sorbent. Contrarily, hematite increased the leachability of DIM by blocking the active sorption sites on clay platelets. Both hematite and clay sorption acted as type-2 (i.e., rate-limiting) sites. In all soils, IMI exhibited lower short-term leachability than DIM. Combined with a body of data concerning other aspects of IMI environmental behaviour, the results indicate that the risk of accumulation of IMI in the soil is greater than the risk of contamination by leaching. Thus, continuous monitoring of IMI in soils should be incorporated into future soil health protection programs.

## 1. Introduction

Neonicotinoids are the most important group of insecticides since the 1990s [1,2,3], registered for use in more than 120 countries worldwide [4,5]. They owe their popularity to a combination of properties like high effectiveness (primarily against aphids, whiteflies, and planthoppers) and long-term protection, but—most of all—for their specific binding to nicotinic acetylcholine receptors of invertebrates, which makes them less harmful to vertebrates [6,7]. Among them, imidacloprid (IMI) was the first one in use and still holds the top positions in sales [4] and consumption, especially in the Americas and Africa [8,9,10].

However, the deleterious effect of IMI on non-target species such as bees has been discovered and thoroughly studied during the last decade [11,12,13,14], which resulted in a complete ban of IMI for all outdoor use in EU states from 31 July 2022 [15,16]. It will remain to be approved for use in permanent greenhouses [17]. Efforts are being made in other countries to follow the EU’s footsteps [2], but with limited success. Reasons for concern keep growing due to the accumulation of evidence regarding IMI toxicity also on mammals and other vertebrates [3,7,18,19,20,21,22,23,24,25].

IMI is often classified as a substance with high leaching potential [7,26,27,28,29,30], although there are reports stating the opposite [31,32], or showing that the leaching potential depends on the formulation [33]. While IMI degrades photolytically, it is stable to hydrolysis at environmentally relevant pH values (i.e., 5 < pH < 9) [7,30]. It was found persistent in water samples and not readily biodegradable in aquatic environments [32]. Half-life in water and water-sediment is 30 and 129 days, respectively, while the half-life in soil ranges from 174 up to 578 days [30]. IMI and other neonicotinoids were detected worldwide in plants and food, as well as surface, underground, and even drinking water [7,26,34,35] in amounts as high as 25,000 times the legal limit [36]. Nevertheless, IMI is still not routinely monitored in soils, despite the fact it is recognized as a contaminant of emerging concern, and moreover, as one requiring special attention [37]. IMI is also listed on the EU commission’s latest Watch List, a list of ten substances or group of substances chosen due to a significant risk they may pose to or via aquatic environment, but for which the data is not yet sufficient [38,39]. A knowledge gap was recently emphasized regarding neonicotinoids’ sorption/desorption properties and the factors influencing them [40].

In Croatia, IMI is the third most common insecticide [41]. Along with several other EU countries, in 2020 Croatia granted emergency authorization for outdoor use of IMI, due to its effectiveness in the protection of sugar beet [42]. It is also very effective against olive fruit fly (*Bactrocera oleae*), the most noxious pest in olive tree plantations [43,44,45,46]. In our previous publication, we analysed the sorption of dimethoate (DIM) and established that the leaching potential was the highest in the soil taken from the olive tree plantation located in the coastal (Mediterranean) part of Croatia [47]. Mediterranean soils typically have very low organic matter (OM) content and the location of plantations near the Adriatic Sea makes them a potential threat for nearby aquatic life [24]. As the length of the Croatian coastline exceeds 1700 km and comprises terrains of different geological backgrounds [48], the physicochemical properties of soils substantially differ among plantations.

Therefore, based on the alarming facts regarding IMI toxicity on non-target species, the frequency of its detection worldwide, the high leaching potential of Mediterranean soils, and the lack of information on IMI behaviour in them, the aim of this study was to: (a) analyse IMI sorption and transport properties in an array of Mediterranean soils since, to the best of our knowledge, there is no data concerning IMI transport parameters in them; (b) compare it to the transport behaviour of DIM, the second most used insecticide in Croatia and heavily used worldwide [41], and (c) analyse correlations of transport parameters to soil physicochemical properties.

Please note that, contrary to legislative concerning water and air, the coherent legal framework for protecting Europe’s soil does not exist. Only recently, in April 2021, the European Parliament approved a resolution on soil protection [49], which expressed the need for the regulation and implementation of continuous monitoring of soil health status. We believe that the results of this study can contribute to policymaking regarding the implementation of continuous monitoring of IMI into future soil health protection programs.

## 2. Materials and Methods

### 2.1. Chemicals, Reagents, and Standards

Aqueous pesticide stock solutions were prepared from analytical grade IMI (≥99.0%) and DIM (≥99.0%), purchased from Dr. Ehrenstorfer (Germany) (Table 1). Final pesticide concentrations were prepared by dilution of IMI (51.8 mg/L) and DIM (93.4 mg/L) with calcium chloride solution (0.01 M; POCH, Gliwice, Poland). Basic physicochemical characteristics and chemical structures of pesticides are given in Table 1. Mobile phase for pesticide analysis consisted of HPLC grade acetonitrile from J.T.Baker (Deventer, Holland) and ultrapure water from a Mili-Q system (Millipore, El Passo, TX, USA). Other chemicals used were listed in our previous publication [47].

### 2.2. Geographical Locations and Soil Samples

**Figure 1:**
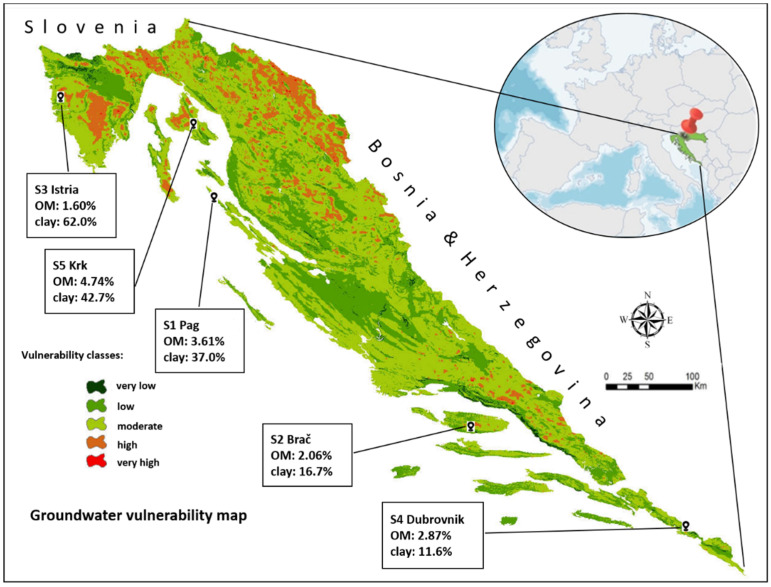
The geographic location of the soil sampling sites and their position on the groundwater vulnerability map, with designated organic matter (OM) and clay content.

The soils were collected from five olive tree plantations distributed in five coastal regions (Istria, Kvarner, North, Central and South Dalmatia) (Figure 1), the location of which is defined by geographic coordinates listed in Table 2.

From each location, soil samples (in five subsamples) were collected from 0- to 30-cm depth of the soil profile, following the standard procedure [44]. The soil samples were air-dried, sieved using a 2 mm mesh diameter sieve, quartered, and mixed in order to obtain one representative soil sample for each location. The collected soils had no residue of previous IMI and DIM use at all, which was confirmed by analyzing their concentration in the soil.

Soil samples (in five subsamples) were collected and their physicochemical characteristics were analysed (Table 2) as described in detail in our previous publication [47].

### 2.3. Pesticide Transport Experiments

Transport of IMI and DIM in the glass columns (column length *L* = 8.0 cm; internal diameter of 10 mm) filled with air-dry soil was analysed according to the OECD procedure [50], as follows: all columns with soil were initially saturated with a background leaching solution (0.01 M calcium chloride, pH = 5.5) at a flow rate of 0.39 mL min^−1^ regulated by a peristaltic pump (Reglo DIG, Ismatec, Switzerland) from the column bottom, in order to determine pore volume (*V*/*V*_0_) of the column. Calcium chloride solution was used to minimize ionic strength changes and to simulate natural pH soil conditions. In the next step, each of the five soil-filled columns was saturated with IMI at the application rate of 2.5 mg kg^−1^. The same column saturation procedure was also performed with DIM. After saturation, all columns were left for 24 h to achieve equilibrium condition. Then, each glass column was continuously washed from the top with background leaching solution at a flow rate of 0.39 mL min^−1^ using a peristaltic pump. Column leachates were periodically collected in 1 mL vials, filtered using rough 0.2 µm nylon filters (Millipore, Bedford, MA, USA) and analyzed directly by HPLC for their pesticide content. Pesticide concentration data were used to generate breakthrough curves (BTCs). Experimental flow conditions and characteristics of each soil column are listed in Table S1. Bromide solution (Br^−^) was used as a non-reactive reference tracer for water flow and hydrodynamic parameters determination.

### 2.4. Instrumentation and HPLC Analysis Conditions

Analyses of the pesticides were performed on a liquid chromatography system (Shimadzu, Japan) equipped with an SPD-M10Avp diode array detector and a C18 column (250 mm × 4.6 mm; Phenomenex, Torrance, CA, USA). Isocratic elution was carried out by mobile phase of acetonitrile/water 50:50 (*V*/*V*) mixture for IMI and 40:60 (*V*/*V*) for DIM under the flow rate of 1 mL/min, and 200 nm (270 nm) absorption wavelength for DIM (IMI), respectively. Retention times were 5.3 min (DIM) and 3.9 min (IMI). Calibration curves were linear up to 10 mg/L with a regression coefficient of *R*^2^ > 0.999 (eight calibration points, in duplicate). The detection limits were 5 µg/L (IMI) and 25 µg/L (DIM). The average recovery was 91.4% (IMI) and 94.7% (DIM), with a relative standard deviation lower than 5%.

### 2.5. Data Evaluation and Statistical Analysis

The fitting and modeling of breakthrough curves was performed using the Wolfram Research Mathematica^®^ Version 10.0 (Champaign, IL, USA) and Stanmod^®^ Version 2.0 (Riverside, CA, USA) with CXTFIT algorithm software using two-site nonequilibrium sorption model (NELM) (Section 2.6). The agreement between experimental and model data was evaluated by the Pearson correlation coefficient (*R*^2^), the Scaled Root Mean Squared Error (SRMSE) and error of *χ*^2^ test. Statistica^®^ software package Version 13 (StatSoft, Inc, Tulsa, OK, USA) has been used for descriptive and correlation analysis of experimental results. All experimental data were presented with the median of three determinations and with minimal and maximal values. Results were considered statistically significant at *p* < 0.01. Due to the small number of experimental results, nonparametric statistical tests were used. Correlations of soil properties with transport parameters were analyzed by the Kendall–Tau statistical test.

### 2.6. Modelling of Column Experimental Data

The two-site nonequilibrium sorption model (NELM) was used to simulate the transport of pesticides in the soil profile. This model was chosen based on its successful application in the research of transport of other pesticides such as atrazine [51]. According to this model, there are two types of sorption sites [52,53,54]: (a) type-1, which assumes that sorption/desorption is instantaneous (equilibrium), (b) type-2, where time-dependent, i.e., rate-limiting sorption processes occur (non-equilibrium). Parameters used in NELM are shown in the following equations:

(1) $$\left(1+\frac{f\rho K_{d}}{\theta_{v}}\right)\frac{\partial \gamma }{\partial t}+\frac{\rho }{\theta_{v}}\frac{\partial S_{2}}{\partial t}=D\frac{\partial^{2}\gamma }{\partial x^{2}}-v\frac{\partial \gamma }{\partial x},$$

(2) $$\frac{\partial S_{2}}{\partial t}=\alpha \left[\left(1-f\right)K_{d}\gamma -s_{2}\right],\;$$

where *ρ* is the bulk density (g/cm), *K*_d_ is the distribution coefficient (L/kg), *Θ*_V_ is the volumetric water content at saturation, *γ* is the solute concentration in the liquid phase (mg/L)*, t* is the time (min), *s*_2_ is the concentration of the solute adsorbed at type-2 sites (mg/L), *D* is the hydrodynamic dispersion coefficient (in cm^2^/min; this parameter combines mechanical dispersion with molecular diffusion), *x* is the distance (cm), v is the pore–water velocity (cm/min). Prominent parameters are: retardation coefficient *R* (dimensionless quantity, indicating the strength of interaction, proportional to *K*_d_); *f—*the fraction of pesticides sorbed at type-1 sorption sites (i.e., population distribution between type-1 and type-2 sites); *α—*first-order rate coefficient (min^−1^) for type-2 sorption. *f* and *α* are calculated from Damköhler number *ω* and the variable *β*, respectively. *ω* is the dimensionless mass transfer coefficient and represents the ratio of hydrodynamic residence time to characteristic time for sorption “reaction”. *β* reflects the distribution of a pesticide between instantaneous (type-1 sites) and rate-limiting (type-2 sites) domains, i.e., the fraction of instantaneous solute retardation. Along with *R*, *ω* and *β* were obtained by fitting the breakthrough curves (BTCs) using the CXTFIT program. BTCs represent relative pesticide concentration (*γ/γ*_0_) in the leachate versus the relative pore volume (*V*/*V*_0_, representing dimensionless time [27,53]). The parameters are related as given in the following equations:

(3) $$R=1+\frac{\rho \cdot K_{d}}{\theta_{v}},$$

(4) $$\beta =\left(1+\frac{f\cdot \rho \cdot K_{d}}{\theta_{v}}\right)/R,$$

(5) $$\omega =\left[\alpha \cdot \left(1-\beta \right)\cdot R\cdot L\right]/v,$$

(6) $$P=\frac{v\cdot L}{D}$$

where *L* is the column length (cm) and *P* is the Peclet number, estimated for each column from tracer (Br^−^) BTC. Tracer BTCs were also used to exclude the existence of transport related non-equilibrium (Section 3.4), a necessary condition for the application of NEL model [51,53]. The tracer exhibited symmetrical BTCs (data not shown), that were successfully fitted with equilibrium sorption model [47,51,53,54], thus justifying the use of NELM for modelling pesticides’ BTCs and allowing determination of hydrodynamic parameters.

## 3. Results and Discussion

### 3.1. Comparison of Chemical Structure and Sorption Isotherms

In order to facilitate the analysis of the BTCs, allow us to first invoke and further analyse the sorption properties of IMI from our previous research, in relation to DIM as the second most used insecticide in Croatia [41,47,55]. We focused on the low concentration range of the sorption isotherms since common concentrations of IMI in realistic soils are 12–18 ppb [56].

The chemical structure of sorbate molecules determines their sorption properties, which in turn govern the transport properties [40,57,58]. Even though the molecules of both insecticides contain polar moieties, their chemical behaviour is quite different, as reflected, e.g., in drastically different water solubilities (Table 1). One of the structural differences is the presence of the pyrimidine aromatic ring in IMI, while DIM is a purely aliphatic compound. Another distinct feature of IMI is the coplanarity of the guanidine and the nitro substituent, a prerequisite for the conjugation that enables a negative charge flow towards the nitro group, making it a negatively charged tip of the molecule (Table 1) [59]. The second partially negative part of the molecule is a nitrogen atom within the pyridine ring. On the other hand, positive charge is not located at a distinct atom but is instead distributed over the guanidine and imidazolidine groups [59]. The nitrogen in the pyrimidine ring can act like a hydrogen bond acceptor, as well as the oxygen in -NO_2_. The guanidine moiety of IMI has p*K*_a_ values of 1.56 and 11.12; hence, the protonation of IMI is negligible at the pH range relevant to soil [6,60].

**Figure 2:**
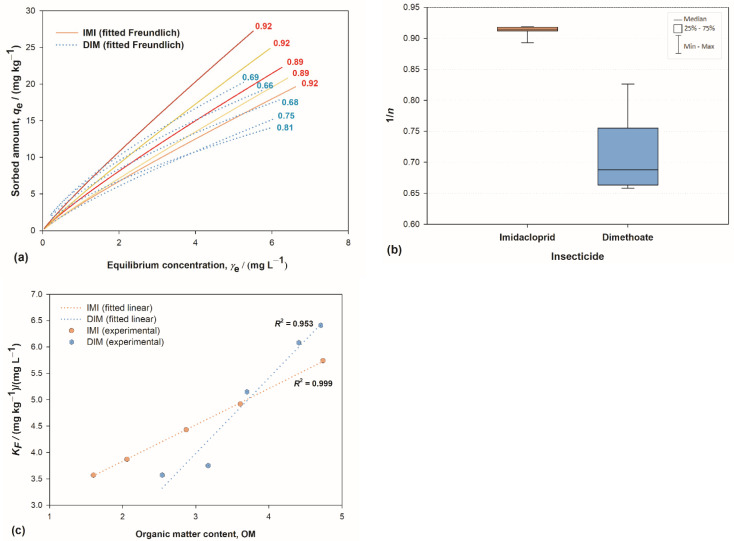
(**a**) Freundlich fits of sorption isotherms for the sorption of imidacloprid (IMI) and dimethoate (DIM) on Croatian soils; numbers indicate 1/n value; (**b**) box-whiskers plot depicting the distribution of 1/n values IMI and DIM; and (**c**) influence of organic matter content on Freundlich sorption coefficient *K*_f_ for IMI and DIM.

Chemical structure differences were reflected in different types of adsorption isotherms. The superposition of IMI’s and DIM’s isotherms (Figure 2a) clearly showed that DIM’s isotherms deviate from linearity more severely than IMI’s. The isotherms were obtained by the Freundlich adsorption model, where the deviation from linearity (the degree of curvature) is quantified by the 1/n parameter and provides information regarding the heterogeneity of sorption sites energies; the parameter *K*_F_ is related to sorption efficiency. 1/n had a value around 0.9 for all IMI isotherms, while for DIM they spanned from 0.66 to 0.81 (Figure 2b), indicating dissimilar sorption mechanisms. According to archetypal Giles’ classification [61], DIM isotherms can be classified as L-curves: nonlinear, with the highest slope at the initial stage. Such a shape indicates more efficient sorption at low solute concentration; the increase of solute concentration hinders further sorption due to the diminished number of empty sorption sites, and the sorption is not cooperative. On the other hand, highly linear IMI sorption isotherms [60,62,63] can be classified as C-curves, which occur when the solute penetrates the solid more readily than does the solvent [61]. There are four general systems consistent with this type of sorption, one of which is the sorption of non-ionic aromatic solutes on hydrophobic sorbent; clearly, the sorption of IMI to soil organic matter (SOM) is compliant with this description. Linearity of the isotherms indicates that the number of sorption sites remains constant (up to the point of complete saturation). This is possible if sorption at one site is accompanied by a creation of additional sites: the first sorbate molecule penetrates the sorbent structure, breaks the bonds within it, and in this way opens the structure, allowing more sorption sites to be created. This mechanism is consistent with previous studies [31,64] that showed an increase in IMI sorption strength with residence time in the soil, attributed to diffusion processes into less accessible or stronger sorption sites in soil.

In a complex matrix such as soil, sorption to other soil constituents cannot be ignored. Clay is one of the most prominent ones, known to have a major impact in sorption of numerous substances [58,65,66,67,68], including DIM [47,69]. The sorption activity of other constituents can be assessed through the 1/n parameter: IMI 1/n values, alongside being larger than DIM’s, are placed in a very narrow range (Figure 2b). This indicated a high degree of homogeneity of sorption site energies for IMI in all soils, regardless of the large differences in their clay content (Figure 1). Note, for instance, that soil S2 and S3 had very similar 1/n values, while they differed in clay content by a factor of four. Such results implied that, unlike for DIM, the clay platelets are not an active sorbent for IMI, even in soils with very large amounts of clay and an average amount of SOM.

These differences can be attributed to the distinct chemical structures of the two molecules, described above. The distribution of partial charges is such that IMI is not prone to interact with negatively charged clay platelets, at least not at typical soil pH values. Only at pH values lower than 1.56, at which IMI assumes its cationic form, the sorption on clay surfaces becomes substantial [60]. On the other hand, the presence of the aromatic pyrimidine ring renders it suitable for relatively strong interaction with SOM, especially with aromatic moieties. The partition of IMI between clay and SOM is thus biased towards the SOM to a much greater extent compared to DIM. This view is consistent with studies that showed that (a) the IMI sorption coefficient is an order of magnitude larger for sorption on pure humic acids compared to the one obtained on pure clay [60,70]; (b) IMI has a strong affinity to the hydrophobic environment [71]; (c) IMI’s pyrimidine aromatic ring tends to be embedded in the environment of aromatic amino acid residues [6], and (d) the neonicotinoids, in general, have the low affinity for soil minerals [72]. Additionally, the results for both pesticides showed a rise in *K*_F_ values proportional to SOM content, as expected [73,74], but the correlation was nearly perfect for IMI and less so for DIM (Figure 2c), which further corroborates the aforementioned conclusions.

A degree to which a pesticide molecule prefers one phase over the other will affect every other aspect of its behavior in the soil [67]. Thus, sorption properties serve as a basis for investigation of IMI transport characteristics via analysis of the breakthrough curves.

### 3.2. Analysis of Transport Properties

**Figure 3:**
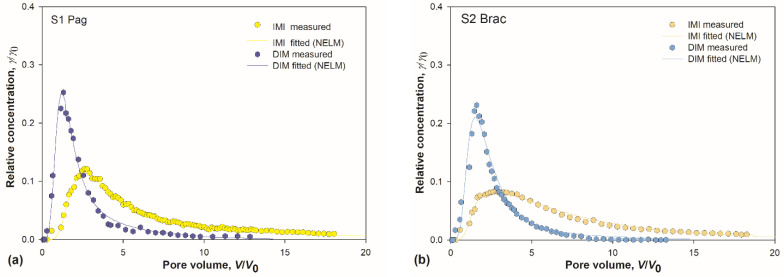

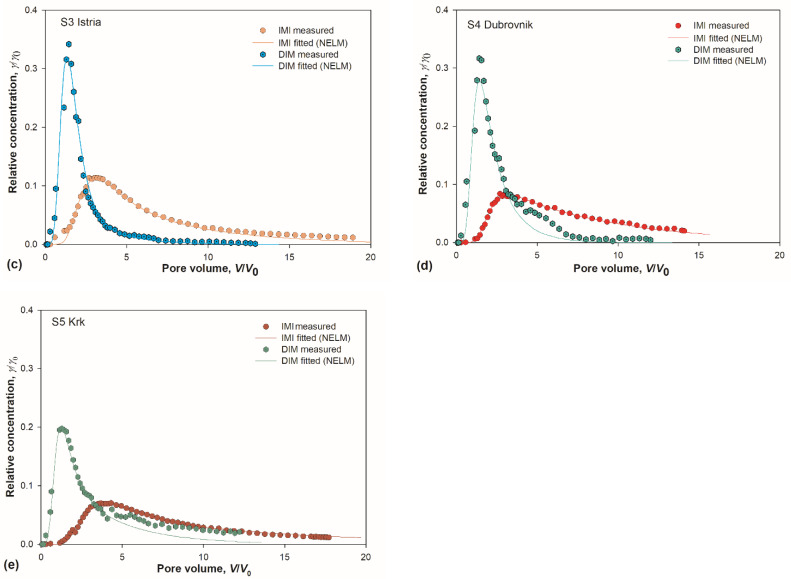

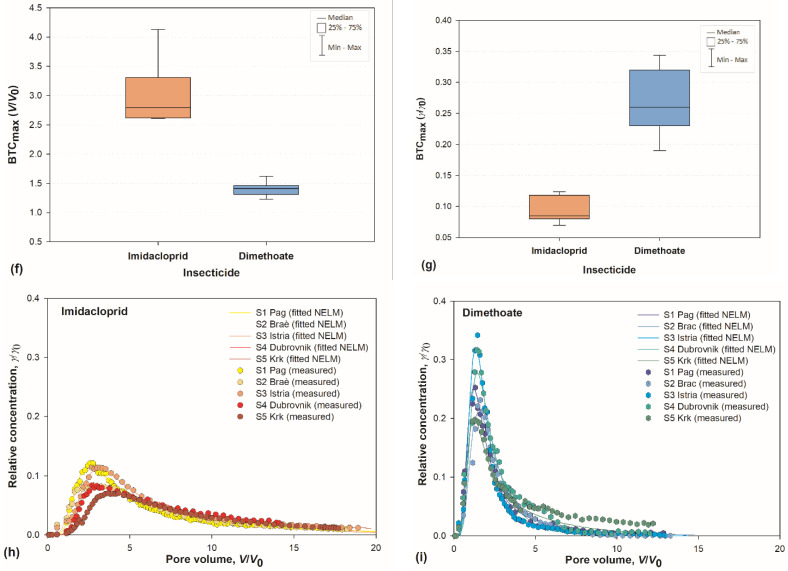
(**a**–**e**) Measured and simulated breakthrough curves (BTCs) for imidacloprid (IMI) and dimethoate (DIM) in soils S1–S5. Experimental points are median value of three measurements. Simulated BTCs were obtained with non-equilibrium transport model (NELM); (**f**,**g**) box-whisker plots depicting distribution and position of BTCs maxima for IMI and DIM; and (**h**,**i**) pooled BTCs for IMI and DIM.

IMI breakthrough curves (BTC) were analysed using the two-site kinetic model [53] and compared to DIM transport behaviour in the same set of soils. Observed and fitted BTCs are shown in Figure 3a–e.

BTC curves of both insecticides were asymmetrical with pronounced tailing, but with prominent differences between them. Generally, IMI curves were more levelled and broader, with maxima shifted toward higher pore volumes. Maxima of all five DIM curves were sharp and located within a narrow range centred around 1.4 *V*/*V*_0_, while the distribution of IMI maxima locations was wider and centred around 2.8 *V*/*V*_0_ (Figure 3f–g). The tailing was markedly pronounced in all IMI curves, as reflected in larger retardation factors (Table 3). It is well known that the tailing reflects the strength of interaction between the sorbent and the sorbate [54].

These core differences between the two groups of BTCs demonstrate that the sorption was more efficient for IMI compared to DIM. The mobility and leaching potential were lower for IMI. Relatively low leaching potential of IMI, compared to two other neonicotinoids, was established by Kurwadkar et al. [75], and was ascribed to its lowest water solubility. Note that water solubilities of IMI and DIM are 0.51 g/L and 39.8 g/L, respectively. Most of the DIM molecules were eluted in the early stages of elution, demonstrating its high leaching potential, which agrees well with our previous results for DIM sorption in a different set of soils [47].

On the other hand, the results are in discrepancy with the high-leachability status of IMI [Groundwater Ubiquity Score (GUS) index > 2.8] against the transition leaching potential of DIM (1.8 < GUS < 2.8) [30,76]. The discrepancy arises from the fact that the GUS index accounts for pesticides’ half-life, which is very large for IMI, thus it aims to describe the leaching potential over a longer duration. Yet, it does not account for the differences in sorption mechanisms of pesticides: the leaching potential of IMI in fact diminishes with residence time in soil [31,64]. Also, the GUS index is calculated from *K*oc, a parameter still regarded as “universal” although known to be inexact [67].

Comparing IMI curves among themselves (Figure 3h), it is apparent that the soil S5 is discernible from the rest due to the lowest maximum located at the highest *V*/*V*_0_. The retardation factor for IMI in S5 was the highest among all the soils (Table 3). Having in mind the conclusions inferred from the sorption isotherms analysis, which indicated that IMI has a strong bias towards OM compared to other soil constituents, these results were ascribed to the highest amount of OM in soil S5. The most pronounced tailing for DIM in the same soil (Figure 3i) was attributed to the same factor, especially when combined with a substantial amount of clay present in S5.

#### 3.2.1. Chromic Luvisol (Terra Rossa)

Istria is a region famous for its olive oil and wine production, and a representative of chromic luvisol soil (i.e., terra rossa) which is widespread over the Mediterranean [77,78], thus we analysed soil S3 in further details.

The BTC for soil S3, with the lowest amount of OM, did not show any pronounced deviation from the rest of the curves for IMI, as might be expected based on low OM. On the other hand, visual evaluation and the retardation factor for DIM indicated that this soil does have the lowest sorption capacity for DIM. This agreed well with our previous study, where Istrian soil was the one with the highest leaching potential for DIM [47]. However, these results might appear discordant with the extraordinary amount of clay, an active sorbent for DIM. The peculiar nature of chromic luvisol (i.e., terra rossa) soils, represented by soil S3, provides a plausible explanation. This reddish soil is characterized by low amounts of OM and large amounts of clay, with red colour originating from the dominance of mineral hematite over goethite. The interaction of hematite and clay can be understood from a series of studies showing a close connection of these two soil constituents and proving that the presence of clay in fact promotes the formation of hematite over goethite [79,80]. It is therefore reasonable to assume that large amounts of hematite hindered the activity of sorption sites on the clay outer surface. This explanation is also corroborated by studies on other pesticides, showing that Fe-oxides decrease sorption by blocking sorption sites on montmorillonite clay [81]. However, the validation of these inferences is needed, together with the characterisation of clay types, which will be analysed in subsequent studies.

Consequently, a new question arose: what are the characteristics of hematite as a sorbent? Chemically, hematite is corundum-type alpha-Fe_2_O_3_. Understanding its surface structure is challenging due to the presence of mixed ionic and covalent bonding, but computer simulation techniques brought a lot of progress [82]. Two surfaces that are the dominant growth faces in natural hematite are {0001} and {0112}. On {0001} surface, the cationic sites are protruding, while {0112} surface contains raised undulating rows of oxygen with surface Fe-cations five-fold coordinated with oxygen [82,83]. Previous studies showed that benzene interacts with {0001} surface through π-bonding with Fe-cations, and even stronger to {0112} surface through direct chemical bond formation between benzene and both kinds of surface atoms [83]. As IMI contains aromatic moiety, it is reasonable to assume that hematite presents an active surface for IMI sorption, although with diminished efficiency compared to pure benzene due to steric effects. Large amounts of hematite, inherent to terra rossa soil, would thus explain the average BTC for the soil S3 containing less-than-average amounts of SOM. On the other hand, the same soil showed the weakest sorption capacity for DIM (Figure 3i), indicating that the non-aromatic nature of DIM renders it impervious to adsorption onto hematite. Chemisorption via oxygen atoms in the phosphinothioyl group would be possible [84], but in DIM the oxygen atoms are methylated, leaving only the carbonyl group as a potential site for weak physisorption. The BTC of DIM for S3 demonstrates that large amounts of clay, also inherent for terra rossa soils, are indeed hindered as an active sorbent for DIM by the presence of hematite. The clay, even in such large amounts, did not improve the leaching potential of terra rossa soil for DIM to any extent.

### 3.3. Two-Site Nonequilibrium Adsorption Model

The modelling of BTCs with the two-site adsorption model yielded an array of informative transport parameters (Table 3), that serve as means of quantification of transport properties and provide deeper insight into transport mechanism. The two-site adsorption model takes into account the fact that the sorption varies by mechanism and by rate for different soil constituents (OM, soil minerals, Fe-oxides, etc.). Sorption sites are classified into two groups: (a) type-1, which assumes that sorption/desorption is instantaneous (weak), and (b) type-2, where slower, i.e., rate-limiting sorption processes occur. Type-2 sorption processes are described by first-order rate coefficient α. Lower value of α indicates slower sorption processes. Partitioning coefficient *f* provides the fraction of molecules undergoing type-1 sorption (i.e., it gives the population distribution between type-1 and type-2 sites). Coefficient *f* is related to retardation factor *R* and rate coefficient *α* as given in Equations (3)–(5). Transport simulations obtained with this model were in good agreement with observed BTCs, albeit less so for DIM, as demonstrated by statistical parameters *R*^2^, SRMSE and *χ*^2^-error, listed in Table 3.

**Figure 4:**
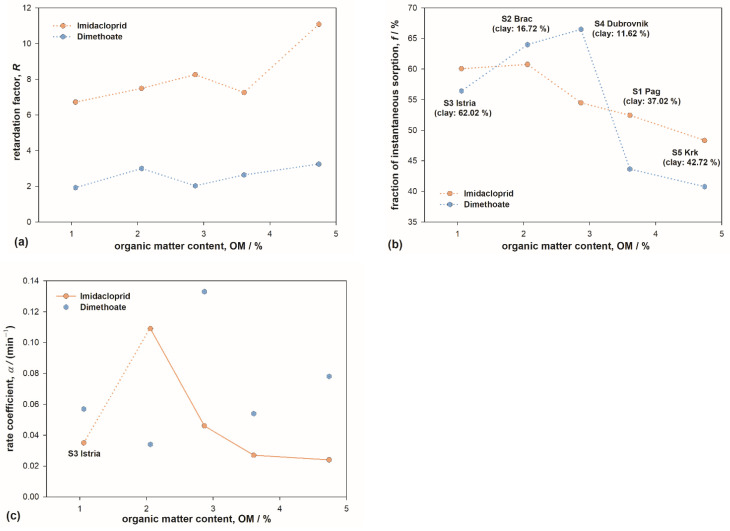
The influence of organic matter content on (**a**) retardation factor *R*; (**b**) partitioning coefficient *f*; and (**c**) first-order rate coefficient *α* for type-2 sorption sites for imidacloprid and dimethoate. The parameters were obtained by modeling pesticides’ breakthrough curves with two-site adsorption model. The lines are guides for the eye.

IMI retardation factors were markedly higher compared to DIM, indicating stronger interaction of IMI molecules with soil constituents, making them less mobile when passing through the soil profile. Differences in the strength of interactions can be attributed to their chemical structure (Section 3.1). For both insecticides, a positive correlation of *R* values with OM content was observed (Figure 4a and Table 4), as could be expected since it is well known that OM content is the dominant feature enhancing sorption properties [70]. The results also showed that the retardation factor increase was more pronounced for IMI, which can be attributed to a stronger preference of IMI towards OM. Retardation factors for IMI obtained here are larger compared to rare other studies [85,86], which can be attributed primarily to higher OM content.

On the other hand, the coefficient *f* showed a decreasing trend with OM content, with protruding values in soils S2 and S4 for DIM (Figure 4b). Since *f* represents the fraction of molecules undergoing weak, instantaneous type-1 sorption, this result indicates that the type-2 sites are more abundant as the OM content increases. For IMI, the decrease was fairly steady (significant negative correlation with OM, Table 4), indicating that type-2 sites are almost exclusively connected to/located at the organic phase. However, for DIM, the results showed markedly larger amounts of type-1 (i.e., smaller amounts of type-2) sorption sites in soils S2 and S4. It is reasonable to attribute this to comparatively small amounts of clay in these soils. This result thus implied that sorption sites on clay platelets can be classified as type-2 sites for DIM. Such interpretation would also explain lower *f* values for DIM compared to IMI obtained for the rest of the soils, containing large amounts of clay.

While parameter *f* provides an estimate of population distribution between type-1 and type-2 sites, coefficient *α* provides information regarding the reaction rate for type-2 sorption. Focusing on IMI (Figure 4c), it can be discerned that–except for soil S3–coefficient α diminishes with increasing OM content. Lower *α* implies a slower sorption/desorption process; thus, this result corroborates the sorption mechanism deduced from IMI sorption isotherms (Section 3.1). Soil S3 showed pronounced deviation from the trend; note that this soil is a representative of terra rossa, a widespread type of soil in the Mediterranean, characterized by large amounts of hematite. The analysis of BTCs, combined with the chemistry of hematite and pesticide molecules, led us to infer that hematite exhibited certain sorption activity for IMI (Section 3.2.1). A markedly lower value of rate coefficient α obtained for soil S3 (Figure 4c) verified those deductions. The value of *α* for soil S3 is close to values in soils containing threefold amounts of OM. Additionally, it is reasonable to assume that hematite is also responsible for the diminished fraction of type-1 sorption sites in S3 (Figure 4b). These two observations also lead to a conclusion that sorption of IMI on hematite can be classified as type-2 sorption.

The values of *α* for DIM are scattered within a similar range, but without any apparent trend. This is most likely a consequence of the coexistence of two kinds of type-2 sorption sites for DIM (OM and clay) combined with the intricate nature of OM/clay clusters that, unlike hematite, exist in all soils [47,67].

Kendall–Tau correlation test also showed insignificant correlation between transport parameters and soil pH. This can be attributed to the fact that pH of the investigated soils ranged from 4.34 to 7.13, while the guanidine moiety of IMI has p*K*a values of 1.56 and 11.12. According to Farenhost [73], and in accordance with our previous results [47,55], the soil acidity has an effect on the sorption of pesticide molecule only if p*K*_a_ values are within two units of soil pH values. Insignificant correlation was also established between transport parameters and CEC, with the exception of a weak negative correlation for IMI’s coefficient *α*. Under assumption (that should be verified by XRD analysis of clay composition) that lower CEC reflects higher amounts of kaolinite and/or vermiculite (as opposed to montmorillonite), i.e., the clays that exhibit stronger sorption for anionic species [58], this negative correlation can be attributed to IMI’s negatively charged nitro group tip (Section 3.1).

### 3.4. Origins of Nonequilibrium Sorption

There are several processes that cause time-dependent nonequilibrium sorption (Appendix A). Due to a specific charge distribution and aromatic nature and based on the results of sorption isotherms and transport parameters obtained by BTC modelling, we argue that intra-sorbent diffusion is the dominant process causing asymmetry in IMI breakthrough curves and determines its transport properties. IMI molecules diffuse the OM matrix, penetrate the sorbent structure, break the bonds within it, and open the structure. Based on molecular docking calculations [71], it is likely that IMI bonds to OM by creating hydrogen bonds through its nitro group with OM functional groups (carboxyl, hydroxyl, amide) and also by π-interactions through its pyridine ring with OM aromatic moieties, that are especially abundant in humic acids. The results of other studies [31,64] suggest that the penetration to deeper layers of organic phase lasts for weeks and enhances IMI sorption strength, thus diminishing its leaching potential with time. When hematite is present in the soil, the retarded intraparticle diffusion mechanism also contributes to IMI sorption. Strong physisorption is probable for hematite sorption sites also, since π-bonding of IMI aromatic moiety to Fe-cations is a plausible sorption mechanism [82].

Transport properties of DIM are also predominantly determined by rate-limited diffusive mass transfer, with both subtypes present: DIM is sorbed by both OM (intra-sorbent diffusion) and clay platelets (retarded intraparticle diffusion). The transport parameters obtained in this study indicated that both kinds of sorption sites are also type-2 sites, but the sorption appears to be weaker and kinetically faster compared to the strong physisorption process(es) IMI is subjected to, causing the differences in the transport behaviour. Overall, these mechanisms resulted in a lower leaching potential for IMI compared to DIM for all soils.

### 3.5. Imidacloprid Accumulation Potential

Silva et al. [87] recently reported research regarding soil contamination by pesticide residues in agricultural soils of 11 EU member states. 76 pesticides residues were analysed and detected as a single residue or a mixture in 83% of the samples. Only 7 out of 76 compounds were detected in >10% of the samples; two of those were glyphosate and its major metabolite aminomethylphosphonic acid (AMPA), due to the popularity of glyphosate-based herbicides and higher application rates compared to other pesticides. Taking a closer look at the chemical structures of the 5 remaining residues, one can notice they were all aromatic compounds. While aromaticity may diminish compounds’ leaching potential (through stronger interaction with OM), as it did with IMI, it seems to increase its potential for accumulation in the soil. When IMI is used in granules or seed treatment, as high as 80–98% of the active ingredient is not absorbed by the crop but remains in the environment [7]. Relatively long half-lives of neonicotinoids additionally increase IMI’s potential to accumulate in the soil. The data lead to a conclusion that, regarding IMI, the risk of accumulation in the soil is higher than the risk of contamination by short-term leaching. Therefore, the soil should be considered as the primary matrix for IMI monitoring and IMI should be listed as one of the pesticides whose continuous monitoring will be implemented and regulated following the EU resolution on soil protection [49].

## 4. Conclusions

Imidacloprid is often described as a pesticide of high leachability. This study was conducted on five soils from the Mediterranean part of Croatia and all of them demonstrated lower short term leaching potential for IMI compared to DIM. The results indicated that IMI molecules showed a high degree of preference for OM over any other soil constituent. Sorption to OM was found to be a time-dependent (type-2) process and a main sorption-related nonequilibrium process dictating IMI’s transport properties. The fraction of IMI molecules undergoing this type of sorption was correlated to OM content. Additionally, our study indicated that hematite, an emblem constituent of terra rossa soil, also contributed to IMI sorption, whereas clay did not show any sorption activity for IMI. Sorption sites on hematite were classified as type-2 sites, with π-bonding of IMI aromatic moiety to Fe-cations as a probable sorption mechanism. Hematite did not show any sorption activity for DIM, but instead acted antagonistically by blocking the otherwise active sorption sites on clay platelets.

In addition to providing quantitative data regarding IMI transport behaviour and new insight to its sorption to hematite, this study indicated that the risk of IMI accumulation in the soil is higher than the risk of contamination by short-term leaching. Accumulation of pesticides seriously damages soil health, by negatively influencing soil species diversity, growth, and reproduction, all the while new evidence regarding IMI’s toxicity to non-target species keep emerging. Thus, our results, combined with a body of data concerning other aspects of IMI environmental behaviour, lead to a conclusion that continuous monitoring of imidacloprid should be incorporated in future soil health protection programs.

## Figures and Tables

**Table 1:** Chemical structure and selected physicochemical properties of imidacloprid and dimethoate. Shaded areas designate the locations of the negative charge; the positive charge is distributed over the guanidine and imidazolidine groups [20].

Properties	Imidacloprid	Dimethoate
Chemical structure	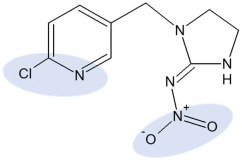	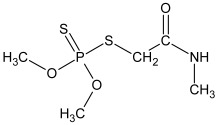
IUPAC name	(*E*)-1-(6-chloro-3-pyridylmethyl)-*N*-nitroimidazolidin-2-ylideneamine	2-dimethoxyphosphinothioylthio-*N*-methylacetamide
Molecular formula	C_9_H_10_ClN_5_O_2_	C_5_H_12_NO_3_PS_2_
Molar mass (g/mol)	255	229
Melting point (°C)	143	43–45
Vapour pressure (mPa)	2.00 × 10^−4^ (20 °C)	0.25 (25 °C)
Water solubility (g/L)	0.61 (20 °C)	39.8 (21 °C)
Log K_OW_	0.57 (20 °C)	0.70 (20 °C)

**Table 2:** Physicochemical properties of the experimental soils.

Parameters	Soil				
	S1	S2	S3	S4	S5
Location	Pag	Brač	Istria	Dubrovnik	Krk
GCS ^1^	44°41′19″ N 14°45′11″ W	43°21′03″ N 16°44′34″ W	45°18′02″ N 13°37′13″ W	42°37′31″ N 18°11′06″ W	45°21′24″ N 14°37′43″ W
Textural classes	clay	loam	clay	sandy loam	clay
Sand	39.3	47.7	19.7	55.8	33.6
Silt	23.7	35.6	18.3	32.6	23.7
Clay	37.0	16.7	62.0	11.6	42.7
pH ^2^	6.42	7.13	4.34	6.74	6.69
HA (cmol/kg) ^3^	5.65	2.98	94.0	4.17	16.2
CEC (cmol/kg) ^4^	34.2	25.7	105	28.0	49.2
Ca^2+^(mg/100 g)	13.0	17.4	7.10	15.8	24.8
Mg^2+^ (mg/100 g)	5.97	1.49	2.35	1.53	3.50
Na^+^ (mg/100 g)	2.24	0.28	0.15	0.24	0.98
K^+^ (mg/100 g)	3.59	3.38	1.20	3.76	2.20
OM (%) ^5^	3.61	2.06	1.60	2.87	4.74
C_oxHa_ (%) ^6^	1.37	0.70	0.53	1.10	1.85
C_oxFa_ (%) ^7^	0.48	0.24	0.19	0.38	0.65

^1^ Geographic Coordinate System; ^2^ measured in soil + 0.01 M calcium chloride mixture (1:2.5 *w*/*V*); ^3^ hydrolitic acidity; ^4^ cation exchange capacity; ^5^ organic matter content; ^6^ carbon of humic acids; ^7^ carbon of fulvic acids.

**Table 3:** Transport parameters obtained by nonlinear transport model describing the imidacloprid and dimethoate breakthrough curves.

Parameters	Soil									
	S1	S2	S3	S4	S5	S1	S2	S3	S4	S5
	Imidacloprid					Dimethoate				
R ^1^	7.26	7.49	6.72	8.26	11.08	2.64	3.00	1.93	2.03	3.25
Β ^2^	0.59	0.66	0.66	0.60	0.53	0.65	0.76	0.79	0.83	0.59
f (%) ^3^	52.5	60.8	60.1	54.5	48.3	43.7	64.0	56.4	66.5	40.8
ω ^4^	0.65	2.38	0.70	1.31	1.04	0.41	0.21	0.20	0.40	0.74
α (1/min) ^5^	0.027	0.109	0.035	0.046	0.024	0.054	0.034	0.057	0.133	0.078
R^2^ ^6^	0.990	0.958	0.976	0.993	0.985	0.946	0.970	0.978	0.954	0.986
SRMSE ^7^	0.079	0.102	0.118	0.057	0.076	0.290	0.192	0.201	0.281	0.176
χ2-error ^8^	7.27	9.15	10.6	5.08	6.85	25.9	17.1	18.2	25.3	16.1

^1^ retardation factor; ^2^ fraction of instantaneous retardation; ^3^ fraction of pesticide molecules sorbed on type-1 sorption sites, i.e., partitioning coefficient; ^4^ Damköhler number (ratio of hydrodynamic residence time to characteristic time of the sorption “reaction”); ^5^ first-order rate coefficients for type-2 sites; ^6^ Pearson correlation coefficient; ^7^ Scaled Root Mean Squared Error; ^8^ error of *χ*^2^ test.

**Table 4:** Kendall-Tau correlation test between soil properties and transport parameters.

Parameters	Soil Properties							
	OM ^1^		Clay Content		CEC ^2^		pH	
	IMI	DIM	IMI	DIM	IMI	DIM	IMI	DIM
*R* ^3^	**0.60**	**0.60**	−0.40	0.00	−0.20	−0.20	0.43	0.43
*f* ^4^	**−0.60**	−0.40	−0.20	**−0.60**	−0.40	−0.40	0.14	−0.05
*α* ^5^	**−0.80**	0.20	−0.40	0.00	**−0.60**	0.20	0.32	0.14

^1^ organic matter content; ^2^ cation exchange capacity; ^3^ retardation factor; ^4^ fraction of pesticide molecules sorbed on type-1 sorption sites; ^5^ first-order rate coefficient for type-2 sites; (N = 15, bold typeface indicates significant correlations with *p* < 0.01).

## Data Availability

Not applicable.

## Supplementary Materials

The following supporting information can be downloaded at: https://www.mdpi.com/article/10.3390/toxics10070358/s1, Table S1: Column packing and experimental flow conditions for imidacloprid and dimethoate transport in experimental soil columns.

## Appendix A

There are several processes that cause time-dependent (rate-limited) nonequilibrium sorption. They are divided into two main groups: transport-related and sorption-related nonequilibrium [88]. Transport-related nonequilibrium originates from spatial or temporal heterogeneities in flow domain, causing concentration gradients that in turn result in diffusive mass transfer (a time-dependent, nonequilibrium process), reflected as asymmetry in BTCs. Based on symmetrical tracer BTCs, this type of nonequilibrium can be excluded as a relevant factor in sorption mechanisms. The second type, sorption-related nonequilibrium, is subdivided into (a) chemical nonequilibrium, when chemisorption occurs and (b) rate-limited diffusive mass transfer within the sorbent matrix, when the dominant process is the physical sorption based on van der Waals interactions. The latter can be further subdivided into (i) retarded intraparticle diffusion and (ii) intra-sorbent diffusion. Retarded intraparticle diffusion signifies a process in which sorbate molecules diffuse within the pores of microporous particles, i.e., mineral soil constituents (clay, sand grains) and undergo sorption to pore walls. In intra-sorbent diffusion the sorbate molecules diffuse into the matrix of the sorbent, i.e., to soil organic matter.
