# Supplementary material for: Emerging Contaminant Imidacloprid in Mediterranean Soils: The Risk of Accumulation Is Greater than the Risk of Leaching
Source: Toxics. 2022 Jun 30;10(7):358. doi: 10.3390/toxics10070358 (PMC9323270; doi:10.3390/toxics10070358)
Supplement: Supplementary file 1 [file toxics-10-00358-s001.zip › toxics-1780635-supplementary.pdf]

Table S1 Column packing and experimental flow conditions for imidacloprid and dimethoate transport in experimental soil columns.

Setup	Parameters	Soil				
		S1 Pag	S2 Brač	S3 Istria	S4 Dubrovnik	S5 Krk
		Imidacloprid				
Column packing	L (cm)[1]	8.00	8.00	8.00	8.00	8.00
	m (g)[2]	12.8	12.1	12.9	11.2	12.6
	ρ (g/cm^3)[3]	1.22	1.52	1.06	1.61	1.09
	Θ_V[4]	0.50	0.45	0.55	0.43	0.52
Input pulse and water flow	γ_0 (mg/L)[5]	5.14	4.91	5.44	4.32	4.96
	Q (mL/min)[6]	0.39	0.39	0.39	0.39	0.39
	v (cm/min)[7]	0.99	1.10	0.90	1.16	0.96
	D (cm^2/min)[8]	1.35	3.00	1.10	1.66	1.12
	Total duration (min)[9]	16.0	15.8	15.2	16.6	16.3
		Dimethoate				
Column packing	L (cm)[1]	8.00	8.00	8.00	8.00	8.00
	m (g)[2]	13.0	13.1	13.4	12.0	12.5
	ρ (g/cm^3)[3]	1.04	1.07	1.02	1.01	1.09
	Θ_V[4]	0.51	0.53	0.54	0.54	0.44
Input pulse and water flow	γ_0 (mg/L)[5]	4.99	5.02	4.97	4.13	4.38
	Q (mL/min)[6]	0.39	0.39	0.39	0.39	0.39
	v (cm/min)[7]	0.97	0.94	0.92	0.92	1.13
	D (cm^2/min)[8]	1.74	1.69	1.00	1.03	2.28
	Total duration (min)[9]	16.7	16.7	17.2	18.6	18.3

[1]column length; [2]mass of dry soil; [3]bulk density; [4]volumetric water content at saturation; [5]input insecticide solution concentration; [6]flow rate; [7]pore water velocity; [8]hydrodynamic dispersion coefficient; [9]time for column saturation
